# Cell-penetrating peptides containing the progesterone receptor polyproline domain inhibits EGF signaling and cell proliferation in lung cancer cells

**DOI:** 10.1371/journal.pone.0264717

**Published:** 2022-03-02

**Authors:** Panthita Kaewjanthong, Sarintip Sooksai, Hironobu Sasano, Gyorgy Hutvagner, Sarah Bajan, Eileen McGowan, Viroj Boonyaratanakornkit

**Affiliations:** 1 Department of Clinical Chemistry and Graduate Program in Clinical Biochemistry and Molecular Medicine, Faculty of Allied Health Sciences, Chulalongkorn University, Bangkok, Thailand; 2 The Institute of Biotechnology and Genetic Engineering, Chulalongkorn University, Bangkok, Thailand; 3 Department of Anatomic Pathology, Tohoku University Graduate School of Medicine, Sendai, Japan; 4 School of Biomedical Engineering, Faculty of Engineering and IT, University of Technology Sydney, Australia; 5 School of Health and Behavioural Sciences, University of the Sunshine Coast, Australia; 6 Sunshine Coast Health Institute, Birtinya, Australia; 7 School of Life Sciences, University of Technology Sydney, Sydney, Australia; 8 Age-related Inflammation and Degeneration Research Unit, Chulalongkorn University, Bangkok, Thailand; Florida International University, UNITED STATES

## Abstract

Non-small cell lung cancer (NSCLC) accounts for the majority (80–85%) of all lung cancers. All current available treatments have limited efficacy. The epidermal growth factor receptor (EGFR) plays a critical role in the development and progression of NSCLC, with high EGFR expression associated with increased cell proliferation and poor prognosis. Thus, interfering with EGFR signaling has been shown to effectively reduce cell proliferation and help in the treatment of NSCLC. We previously demonstrated that the progesterone receptor (PR) contains a polyproline domain (PPD) that directly interacts with Src homology 3 (SH3) domain-containing molecules and expression of PR-PPD peptides inhibits NSCLC cell proliferation. In this study, we investigated whether the introduction of PR-PPD by cell-penetrating peptides (CPPs) could inhibit EGF-induced cell proliferation in NSCLC cells. PR-PPD was attached to a cancer-specific CPP, Buforin2 (BR2), to help deliver the PR-PPD into NSCLC cells. Interestingly, addition of BR2-2xPPD peptides containing two PR-PPD repeats was more effective in inhibiting NSCLC proliferation and significantly reduced EGF-induced phosphorylation of Erk1/2. BR2-2xPPD treatment induced cell cycle arrest by inhibiting the expression of cyclin D1 and CDK2 genes in EGFR-wild type A549 cells. Furthermore, the combination treatment of EGFR-tyrosine kinase inhibitors (TKIs), including Gefitinib or Erlotinib, with BR2-2xPPD peptides further suppressed the growth of NSCLC PC9 cells harboring EGFR mutations as compared to EGFR-TKIs treatment alone. Importantly, BR2-2xPPD peptides mediated growth inhibition in acquired Gefitinib- and Erlotinib- resistant lung adenocarcinoma cells. Our data suggests that PR-PPD is the minimal protein domain sufficient to inhibit NSCLC cell growth and has the potential to be developed as a novel NSCLC therapeutic agent.

## Introduction

Lung cancer is one of the most commonly diagnosed cancers and the leading cause of death worldwide [1]. Lung cancer is classified into two main types, small cell lung cancer (SCLCs) and non-small cell lung cancer (NSCLCs) depending on its molecular pathology. Approximately 80–85% of all lung cancers are diagnosed as NSCLCs [2]. Although there are several therapeutic approaches and drugs used to treat lung cancer most lung cancers will eventually become resistant to these current anticancer drugs, becoming more aggressive and progressing to advanced late stage cancer [3–5]. To-date no anticancer treatment has shown long-term success for patients with NSCLC, thus the need for new therapeutic approaches or drugs for the treatment of NSCLC [6, 7].

The epidermal growth factor receptor (EGFR) is a member of the tyrosine kinase receptor ERBB family and has been shown to play a crucial role in the development and progression of several cancer types [8]. Overexpression of EGFR is frequently found in lung cancer, especially in NSCLCs [9], with previous studies demonstrating a correlation between high EGFR expression and a poor prognosis in NSCLC patients [9–11]. Therefore, EGF signaling has been identified as a desirable targetable pathway to inhibit lung cancer growth. New anticancer drugs targeting EGFR signaling have been developed to treat different types of cancer, including the EGFR tyrosine kinase inhibitors (TKIs), which directly inhibit EGFR tyrosine kinase activity and are currently being used for treating locally advanced or metastatic NSCLC [12–14]. However, the majority of NSCLC patients eventually develop resistance to TKI, hence, the need to develop novel classes of drugs targeting EGFR for more efficacious long-term treatment of NSCLC and other types of cancer.

EGFR signaling starts with EGFR dimerization, autophosphorylation, and sequential stimulation of downstream mitogen-activated protein kinase (MAPK) signaling and other pathways [15, 16]. Tyrosine-residue phosphorylation results in the recruitment of the growth factor receptor-bound protein 2 (Grb2) and human son of sevenless (hSOS) proteins [17, 18]. The Grb2 protein contains a Src homology 3 (SH3) domain which preferentially interacts with a PXXPXR motif or polyproline domain (PPD) within other signaling molecules, including hSOS [19, 20]. The binding of Grb2-SH3 to its ligand, hSOS-PPD, mediates activation of several downstream signaling pathways such as the MAPK, phosphatidylinositol-3-kinase and protein kinase B (PI3K/AKT), and the Janus kinase and signal transducer and activator of transcription (JAK/STAT), leading to an increase in cell proliferation and a decrease in apoptosis [21]. We previously showed that the progesterone receptor (PR) contains a PPD at the N-terminal region that directly interacts with the SH3 domain of various cytoplasmic signaling molecules, including the c-Src tyrosine kinases. The PR-SH3 domain interaction mediates rapid progestin-dependent activation of c-Src and its downstream signaling in mammalian cells [19, 22]. Clinical data suggests that NSCLC patients whose tumors express PR have a higher survival rate than those with no or low PR expression [23], but how the presence of PR improves patient survival rate remains unknown.

We recently demonstrated that PR expression inhibited EGF-mediated signaling and cell proliferation in a NSCLC cell model [24]. A mutation in the PR-PPD, which blocked PPD-SH3 interaction, abolished PR-mediated inhibition of EGFR signaling [24]. Therefore, in this study, we extended our analysis to determine whether the presence of PR-PPD alone was sufficient to mediate PR growth inhibition. To aid the transport of PR-PPD into the cell we added the cell-penetrating peptide (CPP) sequence to the PR-PPD, which enhances delivery across the plasma cell membrane; CPPs are short peptides (5–30 amino acids) that can cross the plasma membrane without a specific receptor. This technique is widely used in drug delivery, which enables the transport of small molecules, nucleic acids, proteins, siRNA, and imaging agents into cells [25–28]. Several CPPs can affect normal cell viability, therefore we chose Buforin 2 (BR2), which is less toxic, has a strong penetrating ability whilst being able to discriminate between normal cells and cancer cell lines [29]. The BR2 peptide can internalize into the target cell by electrostatic interaction with ganglioside, a molecule often expressed on the cell membrane of cancer cells more so than that of normal cells. In this study, we designed the cancer-specific peptides BR2-PRPPD to target NSCLC cell proliferation. CPP peptides used in this study were produced in a yeast *Pichia pastoris* (*P*. *pastoris)* expression system. *P*. *pastoris* is a fast growing, cost-effective expression system with high-yield expression [30]. It secretes peptides directly into the medium, allowing for easy purification [31, 32]. Our results demonstrate the specificity and ability of BR2-PRPPD peptides to inhibit EGF signaling, NSCLC cell growth inhibition, and provide a promising anticancer peptide for NSCLC treatment.

## Materials and methods

### Cell culture

The A549 cell line was obtained from the American Type Culture Collection (ATCC). The NSCLC A549, is a human non- small cell lung carcinoma cell line and has wild-type EGFR expression. A549 cells were cultured in Roswell Park Memorial Institute medium (RPMI; (Gibco/Life Technologies, Gaithersburg, USA) plus 10% fetal bovine serum (FBS; Merck Millipore, Darmstadt, Germany) and 1% Penicillin Streptomycin (PenStrep; HyClone Laboratories, Logan, USA). Human lung epithelial BEAS-2B cells were obtained from the American Type Culture Collection (ATCC). BEAS-2B cells were cultured in Bronchial Epithelial Cell Growth Basal Medium (BEBM) supplemented with BEGM Bronchial Epithelial Cell Growth SingleQuots^TM^ (Lonza, Walkersville, USA). Spontaneously transformed Human Keratinocyte Cell Culture (HaCaT) were purchased from Cell Lines Service (CLS, Heidelberg, Germany). HaCaT cells were cultured in Dulbecco’s Modified Eagle Medium (DMEM; (Gibco/Life Technologies, Gaithersburg, USA) with 10% FBS and 1% PenStrep. The EGFR- mutant PC9 cells were gifted by Prof.Hironobu Sasano (Department of Pathology, Tohoku University Graduate School of Medicine, Sendai, Japan). To generate Gefitinib- and erlotinib-resistant cell lines, PC9 cells were cultured with increasing concentrations of Gefitinib or Erlotinib; 10 nM (2 months), 1 µM (2 months) and 5 µM (2 months). The parent PC9-6M cells were also cultured for 6 months in regular medium to eliminate the effects of long‐term cell culture as previously described [33]. PC9-6M, PC9-GR and PC9-ER cells have EGFR mutations as follows: PC9-6M and PC9-GR: exon 19 deletion; PC9/ER: exon 19 deletion, L858R mutation, and T790M mutation. PC9-6M, PC9-GR and PC9-ER were maintained in 10%FBS-RPMI medium plus 1% PenStrep. Cells were cultivated in a humidified tissue culture incubator at 37°C with 5% CO_2_ and were routinely tested for mycoplasma (Bioneer, Korea).

### DNA cloning and transformation

Nucleotide sequence of peptides were designed as 6XHis-tagged recombinant peptides between the sequences of PR-PPD and buforin2. Eco R*I* and Pst *I* restriction sites were added to the N-terminal and C-terminal of peptide sequences, respectively. These sequences were synthesized and cloned into the pUC19 plasmid vector (GeneArt/Thermo Fisher, USA). Purified BR2-PPD, BR2-2xPPD and BR2-2xΔPPD peptides were ligated into the modified pPICZαA expression vector (Invitrogen/Thermo Fisher, USA) and transformed into DH5α competent *E*. *coli* (New England Biolab, USA). Cells were grown on low salt LB agar plates containing 25 µg/ml Zeocin (Invitrogen/Thermo Fisher, USA) and incubated at 37°C overnight. pPICZαA recombinant plasmids were extracted and purified by Plasmid Maxi kits (Qiagen, Germany).

Purified recombinant plasmids were linearized by Sac *I* and transformed into the *P*. *pastoris* strain KM71H competent cells by electroporation. Cells were selected on YPD plates containing 100 μg/ml zeocin. Yeast genomic DNA were extracted (Zymo research, USA) and further identified by PCR using 5’AOX (GACTGGTTCCAATTGACAAGC) and 3’AOX (GCAAATGGCATTCTGACATCC) primers. PCR products were analyzed by electrophoresis compared with *P*. *pastoris* containing empty pPICZαA.

### CPP peptide expression and purification

To prepare a starter culture, fresh single colonies of *P*. *pastoris* containing the CPP-peptide expression plasmid were inoculated into 10 ml of BMGY-buffered glycerol-complex medium and growth overnight at 30°C with shaking at 280 rpm until the OD_600_ reached 2–6. Then 10% of the starter culture was inoculated in 100 ml of BMGY medium in a 250 ml baffle flask for 24 h. Cells were collected by centrifugation and the pellets resuspended in 20 ml of BMMY-buffered methanol-complex expression medium and incubated at 30°C with shaking at 280 rpm overnight. Cells were harvested by centrifugation at 5,000×g for 5 minutes at 4°C. The supernatant was transferred to a sterile tube and stored at -80°C. The 6xHis-tag recombinant peptides were purified by nickel affinity chromatography (Histrap Fast Flow column, GE) using AKTA start with binding buffer (20 mM Sodium Phosphate buffer pH 7.4, 0.5 M NaCl, and 20 mM imidazole). Peptides were eluted using the elution buffer (20 mM Sodium Phosphate buffer pH 7.4, 0.5 M NaCl and 500 mM imidazole). Each fraction was collected and dialyzed to remove imidazole in phosphate-buffered saline (PBS) pH 7.4 overnight, using SnakeSkin Dialysis Tubing (Bio-Rad, Hercules, USA). Peptide concentrations were determined by Bradford assay (Bio-Rad, Hercules, USA) and resolved on 18% SDS-PAGE gels. After electrophoresis, gels were stained with Coomassie blue staining (Invitrogen/Thermo Fisher, USA). For immunoblotting, one microgram of peptide was loaded into a 4–20% Tris-glycine gel (Bio-Rad, Hercules, USA), transferred to a polyvinylidene fluoride (PVDF) membrane and probed with a mouse monoclonal 6xHis tag antibody [HIS.H8] (1:1000, Abcam, UK).

### CPP peptide intracellular localization

To compare the cellular internalization of peptides between A549 lung cancer cells and non-cancerous human keratinocyte HaCaT cells, A549 and HaCat 2x10^5^ cells were cultured on sterile glass coverslips in 6 well plates in 2% dextran-coated charcoal-treated fetal bovine serum (DCC-FBS)-RPMI medium overnight. Then, cells were treated with 2.5 µM BR2-2xPPD in serum-free-RPMI medium for 1 h and compared to untreated cells. Cells were washed and fixed with 4% Paraformaldehyde (PFA) for 20 min. Fixed cells were washed in 1xPBS 3 times and incubated with 0.5% Triton x-100 for 10 min. After washing, cells were blocked with 1%FBS-PBS for 1 h. Then, cells were incubated with the mouse monoclonal 6xHis tag antibody [HIS.H8] (1:200 v/v, Abcam, UK) at 4°C, overnight. The following day, cells were incubated with Goat anti-Mouse IgG (H+L) Secondary Antibody, Alexa Fluor 568 (1:2500 v/v in 1% BSA-PBS) for 1 h at room temperature in the dark. After washing 3 times in 1xPBS cells were incubated with Hoechst (DNA stain) in 1xPBS for 10 min and further washed with PBS. The coverslip was mounted with a drop of Prolong Antifade mounting medium (Invitrogen/Thermo Fisher, USA), sealed with nail polish, and visualized under a confocal microscopy.

### Cell viability assay (MTT assay)

A549 cells were plated in RPMI supplemented with 1% DCC-FBS (Gibco/Life Technologies, Gaithersburg, USA) and 1% PenStrep at 6,000 cells per well in a 96-well culture plate and incubated for 24 h to achieve 80% confluence. For HaCaT, cells were plated at 8,000 cells per well in 10%FBS-DMEM. The following day, the cultured medium was removed. To activate EGF-induced cell growth, cells were treated with EGF 50 ng/ml (Sigma-Aldrich, St. Louis, USA) compared to the combination of EGF and increasing dose of peptides (0, 0.625, 1.25 and 2.5 µM). BEAS-2B 8,000 cells were plated in serum-free BEGM medium for 24 h and subsequently treated with increasing dose of peptides (0–2.5 µM). Cells were incubated at 37°C with 5% CO_2_ for 24 h. MTT solution (5 mg/ml) (Invitrogen/Thermo Fisher, USA) was added to the cells and incubated for 4 h. The insoluble formazan was dissolved with 10% SDS. Then, absorbance was measured at 570 nm using a microplate spectrophotometer (Biotek Synergy Mx microplate reader, Biotek, USA). To determine the effect of EGFR-TKIs and peptides on EGFR-mutant NSCLC cell proliferation, Gefitinib/Erlotinib sensitive NSCLC (PC9-6M) and Gefitinib or Erlotinib resistant NSCLC (PC9-GR and PC9-ER, respectively) were plated at 8,000 cells per well in 10% FBS-RPMI for 24 h. Cells were then treated with Gefitinib or Erlotinib alone or in combination with increasing concentration of CPP-PPD peptides (0–2.5 µM) for 72 h and analyzed for cell viability using the MTT assay. All experiments were carried out in quadruplets and data was calculated from three independent experiments as mean ± SEM. Results are presented as % cell viability compared with the control value of each experiment.

### Protein extraction and Western blot analysis

A549 2x10^5^ cells were cultured in 2% DCC-RPMI medium for 24 h. Cells were pre-treated with BR2-2xPPD or BR2-2xΔPPD 2.5 µM for 4 h, then induced with EGF 20 ng/ml for 5, 10, and 30 min to activate MAPK signaling. Cells were washed with cold PBS and lysed using lysis buffer (5 mM NaF, 2 mM Na_3_VO_4_, 1xproteinase inhibitor in RIPA buffer). Cell lysates were mixed with 4x Laemli loading buffer, loaded on 8% SDS-PAGE gels and transferred onto PVDF membranes.Membranes were probed with primary antibodies recognizing phospho-p44/42 MAPK (1:1000 v/v, Cell Signaling Technology, USA) and total MAPK antibody (1:1000 v/v, Cell Signaling Technology, USA) overnight at 4°C. Next, blots were incubated with an anti-rabbit secondary antibody (1:5000 v/v in 5%BSA-TBST) for 1 h at room temperature and visualized by chemiluminescence using Pierce® ECL Immunoblotting Substrate (Thermo Scientific, USA). Images were analyzed by ImageJ software. All Western Blots shown were representative of at least three independent experiments.

### RNA extraction and real-time PCR

A549 (2x10^5^ cells) were plated in 10%FBS-RPMI medium for 24 h in 6-well culture plates. Then, cells were treated with BR2-2xPPD 2.5 µM for 24 h compared with BR2-2xΔPPD treatment. Cells were wash with cold-PBS, and total RNA was extracted using GENEzol reagent (GeneAid, Taiwan). cDNA was synthesized by adding 1 µg RNA to Accupower RT Premix (Bioneer, Korea) following by real-time PCR with specific primers for cyclin D1 and CDK2. GAPDH was used as a control. Values represent relative gene expression normalized with GAPDH, and mean± SEM calculated from three independent experiments.

### Cell cycle analysis

A549 were seeded in 6 cm dish at 2x10^5^ cells in 10%FBS-RPMI medium. After 24 h cells were treated with BR2-2xPPD or BR2-2xΔPPD 2.5 µM for 24 h compared to untreated cells or vehicle control. A549 cells were collected by trypsinization, washed with 1xPBS, and fixed with cold 70% ethanol at -20°C for at least 3 h. The fixed cells were washed by 1xPBS, centrifuged and the cell pellet resuspended in 200 µl of Muse^TM^ Cell Cycle Reagent (Merck Millipore, Germany) in the dark for 30 mins. The percentage of cells in the G0/G1, S, and G2/M phases were analyzed using the BD FACSCalibur ^TM^ flow cytometer (BD Bioscience, Heidelberg, Germany). Data was collected from groups of at least 10,000 cells and calculated from three independent experiments.

### Statistics analysis

The statistical analysis was determined by employing a paired t-test and a two-way ANOVA with Bonferroni correction using GraphPad Prism 8.0 (GraphPad Software, CA, USA). All data was represented as the mean± SEM of at least three independent experiments and P values of < 0.05 were considered significant in all studies. The symbols (*), (**), (***) and (****) indicate P values ≤ 0.05, ≤ 0.01, ≤ 0.001 and ≤ 0.0001, respectively.

## Results

### Peptide design, expression, and purification

**Fig 1:**
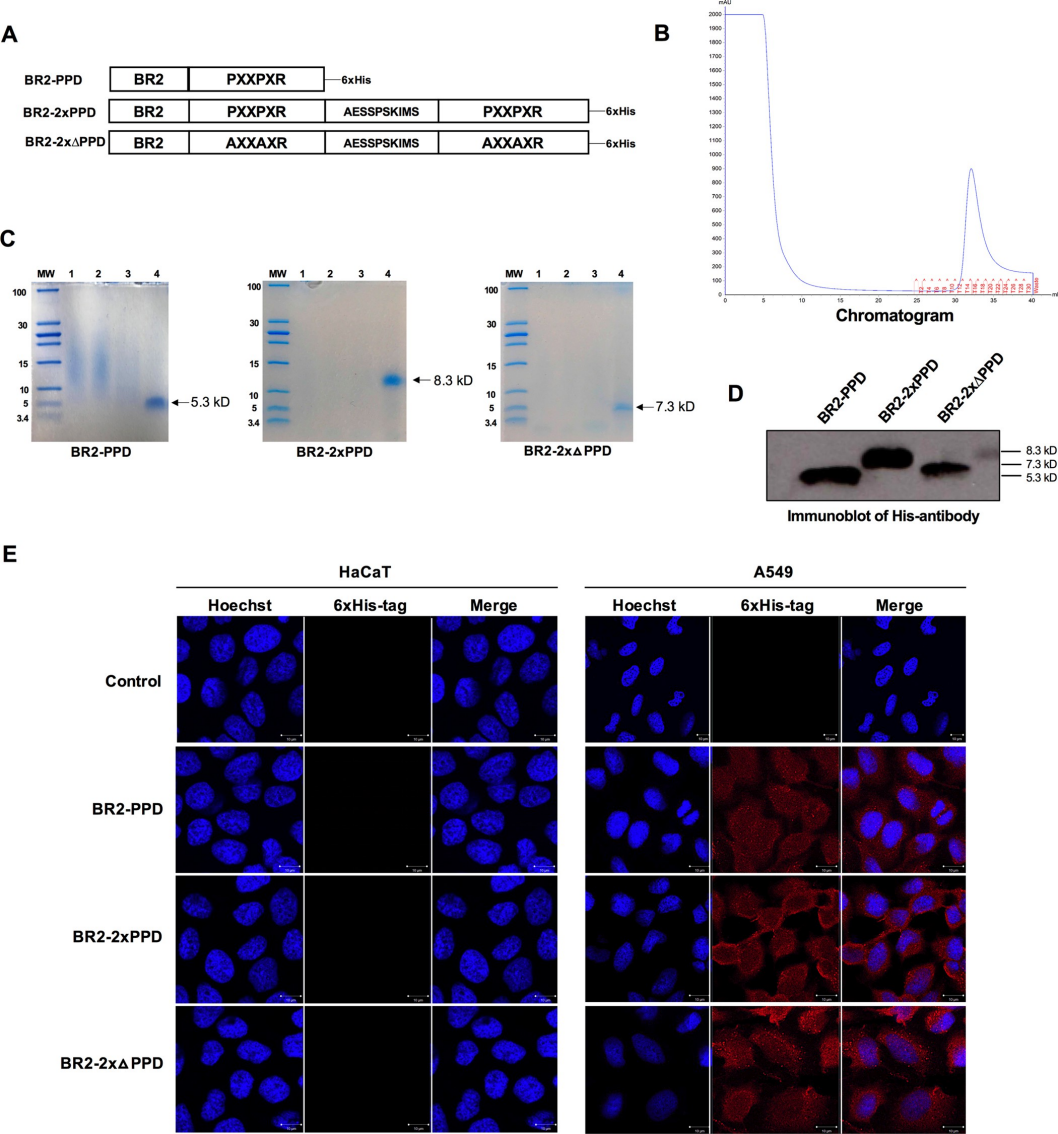
Characterization of the cancer-specific BR2 containing PR-PPD peptides. (A) A schematic of BR2 containing PR-PPD peptides. Polyproline domain (PPD) of human progesterone receptor (PR) was added to the C-terminal of the cell-penetrating peptide (CPP), Buforin2 (BR2). The nucleotide sequences were cloned into a pPICZαA expression vector and transformed into *Pichia pastoris* KM71H by electroporation. All peptides were expressed as Histidine-tagged peptide and purified using a nickel column (B). His-tagged peptides were separated by SDS-PAGE followed by Coomassie blue staining (C) and immunoblotting (D). (E) A549 NSCLC cells and normal HaCaT keratinocyte cells were incubated with His-tagged peptides (2.5 μM) for 1 h at 37°C and stained with a 6-His specific monoclonal antibody. Intracellular localization of His-tagged peptides was visualized by confocal microscopy.

Binding of the SH3 domain to its ligand is essential to transmit signals in several different signaling cascades, which are required for cell growth and several other important biological functions [34, 35]. We previously identified the consensus proline-rich motif PPPPLPPR, a class II SH3 ligand (PXXPXR), within the N-terminal domain of human PR [19]. This PR proline-rich domain (PPD) interacts with SH3-domain-containing proteins and sequentially exerts progestin- activation of nongenomic signaling pathways in mammalian cells [19]. The binding of SH3 domains to their ligands are essential for several growth factor signal transduction pathways, including EGFR. We have shown that expression of PR-B containing the PPD inhibited EGF-induced NSCLC cell proliferation both in the presence and absence of progestin, suggesting that expression of the PR-PPD suppressed cytoplasmic/membrane signaling through interfering with the EGFR signaling pathway [24]. To directly determine whether the PR-PPD is the minimal domain required to mediate the growth inhibitory effect a cancer-specific cell-penetrating peptide PR-PDD peptide was constructed and expressed in a yeast expression system. The cancer-specific CPP, buforin-2 (BR2) [36] was added to the N-terminus of PR-PPD (BR2-PPD) to aid in the delivery of the PR-PPD inside the cells. In addition, BR2-2xPPD was constructed, carrying two repeats of the PR-PPD separated by an intervening sequence similar to a sequence separating the two PPD sequences in hSOS (NP_001369324.1) to mimic endogenous Grb2-SOS interactions. A mutant BR2-2xΔPPD peptide was also constructed to determine the specificity of the PR-PPD interaction by replacing three key prolines with alanines (Fig 1A). All peptides were expressed as 6Xhistidine-tagged peptides in the yeast, *P*. *pastoris* strain KM71H.Transformant yeasts containing the recombinant plasmid with the BR2-PR-PPD sequence were grown in medium containing 0.5% methanol to induce the AOX1 promoter and sequentially secreted BR2-PR-PPD peptides into the culture medium. The supernatant was further purified using nickel affinity chromatography. Histidine tagged peptides were separated and eluted as a single peak shown in Fig 1B, which yielded 100–300 μg of pure recombinant BR2 peptides.

### Characterization of BR2 containing PR-PPD cell-penetrating peptides

To assess the purity of the peptides, the supernatant, flow-through, wash fraction and the eluted peptide were analyzed by SDS-PAGE. We successfully produced highly purified His-tagged CPP PR-PPD peptides as shown as a single band on SDS-PAGE with Coomassie blue staining (Fig 1C) and immunoblotting (Fig 1D). To determine the intracellular localization of the peptides, A549 NSCLC cells were incubated with 2.5 µM of BR2-PPD peptides for 1 h. Cells were fixed and stained with monoclonal antibody recognizing the 6-His synthetic peptide and viewed by immunofluorescence. As shown in Fig 1E, the BR2-PPD, BR2-2xPPD and BR2-2xΔPPD peptides were able to penetrate cell membranes and localize intracellularly in both the cytoplasm and nucleus (red color) as compared to untreated cells. However, these cancer-specific peptides failed to penetrate non-transformed keratinocyte, HaCaT, cells when incubated at equal concentration and time, as shown in Fig 1E. Together, these data suggest that the BR2-PPD peptides possess cancer-specific penetration activity, which are similar to results obtained from BR2-peptides in previous studies [36, 37].

### BR2 containing PR-PPD peptides inhibited EGF-induced cell proliferation

**Fig 2:**
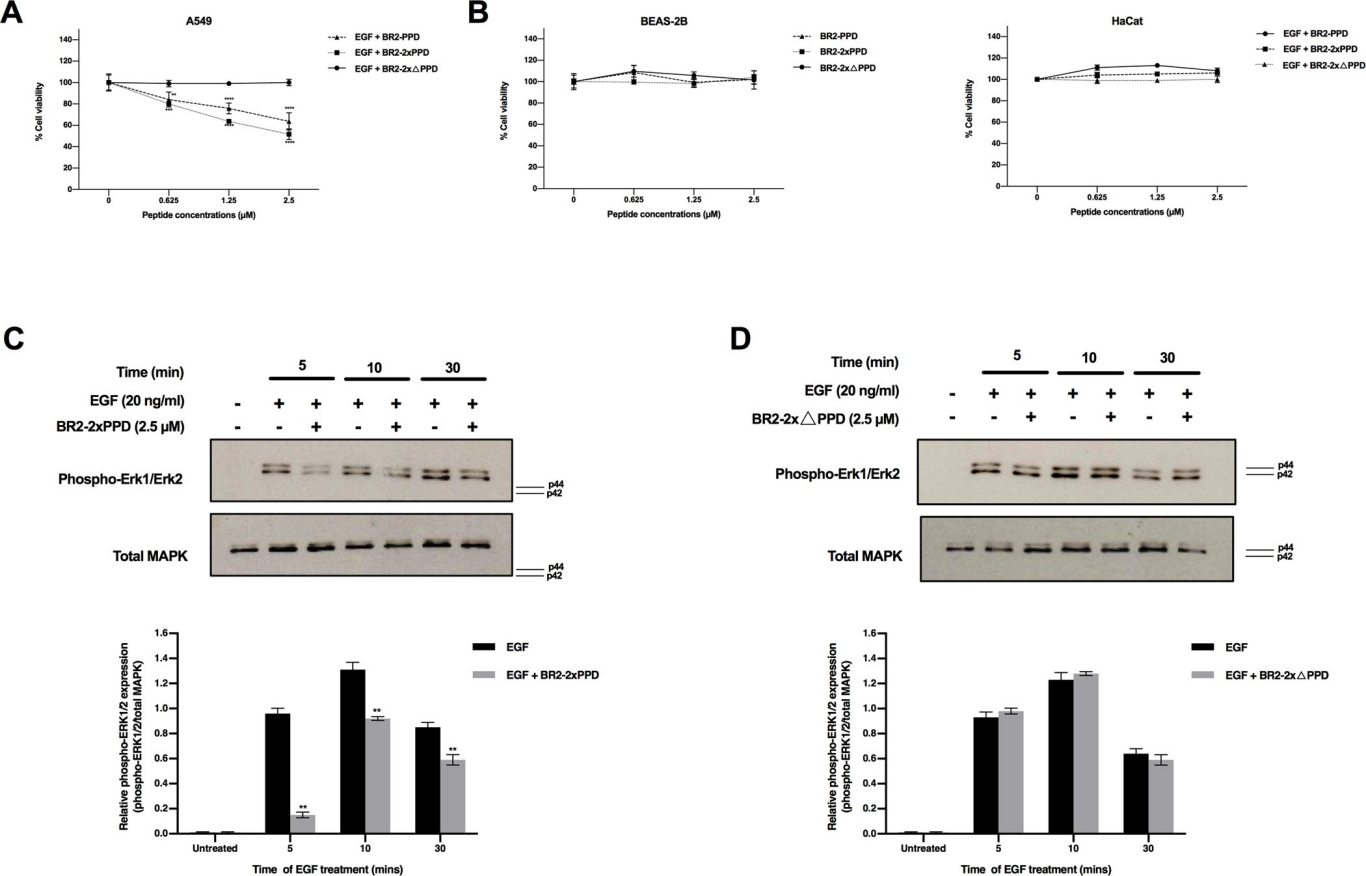
Effect of BR2-PR-PPD peptides on EGF-induced cell proliferation. (A) A549 cells were plated in RPMI supplemented with 1% DCC-FBS and 1% PenStrep at 6,000 cells per well in a 96-well culture plate and incubated for 24 h. The following day, the culture medium was removed. A549 were treated with EGF 50 ng/ml compared to the combination of EGF 50 ng/ml with increasing peptides (0, 0.625, 1.25 and 2.5 μM) for 24 h. Cell viability was analyzed by MTT assays. Cell viability of A549 treated with BR2-PPD, BR2-2xPPD peptides was significantly decreased than BR2-2xΔPPD (control) (**p ≤ 0.01) (***p ≤ 0.001) (****p ≤ 0.0001). Error bars represent the standard errors of the mean (n = 3). (B) BEAS-2B 8,000 cells were plated in serum-free BEGM medium. After 24 h, cells were treated with increasing dose of peptides (0, 0.625, 1.25 and 2.5 μM). HaCaT cells were cultured in 1% DCC-RPMI plus 1% PenStrep at 8,000 cells per well and incubated for 24 h. Next, cells were treated with EGF 50 ng/ml or combined with increasing concentration of peptides (0, 0.625, 1.25 and 2.5 μM) for 24 h. Cell viability was analyzed by MTT assays. (C) and (D) A549 2x10^5^ cells were cultured in 2% DCC-RPMI medium for 24 h. Cells were pre-treated with BR2-2xPPD or BR2-2xΔPPD 2.5 µM for 4 h. Then, cells were induced with EGF 20 ng/ml for 5, 10 and 30 min to activate MAPK signaling. Phospho-Erk1/2 and total MAPK were recognized by western blotting compared to untreated cells. Ten micrograms of protein were loaded in each lane. Bar graphs show relative pMAPK activities (pMAPK/totalMAPK) (****p ≤ 0.0001) and data are shown as means ± SEM (n = 3).

Transactivation of EGFR by cognate ligands such as epidermal growth factor (EGF) promotes NSCLC cell proliferation through PPD and SH3 domain interaction and eventually induces a downstream cascade such as MAPK signaling [19]. To determine whether the presence of the PR-PPD could inhibit EGF-mediated NSCLC cell growth, A549 NSCLC cells were treated with 50 ng/ml EGF alone or in combination with 0–2.5 µM of the BR2-PPD, BR2-2xPPD, or BR2-2xΔPPD peptides. As shown in Fig 2A, the BR2-PPD peptides dose-dependently inhibited EGF-induced A549 cell proliferation with maximum growth inhibition of 36% (±8%) when cells were treated with 2.5 μM of BR2-PPD compared with cells treated with EGF alone. Interestingly, the addition of BR2-PRPPD peptides containing two repeats of the PR-PPD (BR2-2xPPD) was more effective in inhibiting NSCLC proliferation and showed a maximum growth inhibition by 48% (±5%) inhibition. Mutations in the PR-PPD domain, in the mutant BR2-ΔPPD peptide, failed to mediate this inhibition. Importantly, the BR2 containing PR-PPD peptides did not affect the growth of normal bronchial epithelial BEAS-2B and untransformed keratinocyte HaCaT cells at all concentrations tested (Fig 2B). Our data suggested that the PR-PPD is the minimum PR domain required to inhibit NSCLC cell proliferation, and BR2 containing PR-PPD peptides effectively suppressed EGF-induced NSCLC cell, with little to no effect on noncancerous cells.

The MAPK or Erk1/2 signaling pathway is involved in cell proliferation, differentiation, cell survival, apoptosis, and metastasis [38]. Dysregulation of MAPK is often found in advanced cancer, including NSCLC [39]. Our results showed that the BR2-2xPPD peptides effectively inhibited EGF-induced A549 cell proliferation. We next investigated whether the BR2-2xPPD peptide treatment could block EGF-mediated cell proliferation through inhibiting Erk1/2 activation. A549 were pre-treated with 2.5 µM of the BR2-2xPPD peptide for 4 h, followed by EGF treatment at 20 ng/ml for 5, 10, and 30 mins. Phosphorylation of Erk1/2 and total MAPK in cells treated with EGF and the BR2-2xPPD peptides were determined by immunoblotting and compared to cells treated with EGF alone. As shown in Fig 2C, the activation of Erk1/2 was significantly increased when cells were treated with EGF (20 ng/ml) for 5, 10, and 30 mins compared to untreated cells. Importantly, Erk1/2 activation was significantly reduced in all time point tested when cells were pre-treated with the BR2-2xPPD peptide. In contrast, pre-treatment with the BR2-2xΔPPD peptides had little to no effect on EGF-induced MAPK activation at all time tested (Fig 2D). Together, our data suggested that the introduction of the PR-PPD CPP inhibited EGF-induced NSCLC cell proliferation by specifically blocking EGF activation of the MAPK signaling pathway [24].

### BR2-2xPPD is a novel growth-inhibitory peptide for NSCLC expressing wild-type EGFR

**Fig 3:**
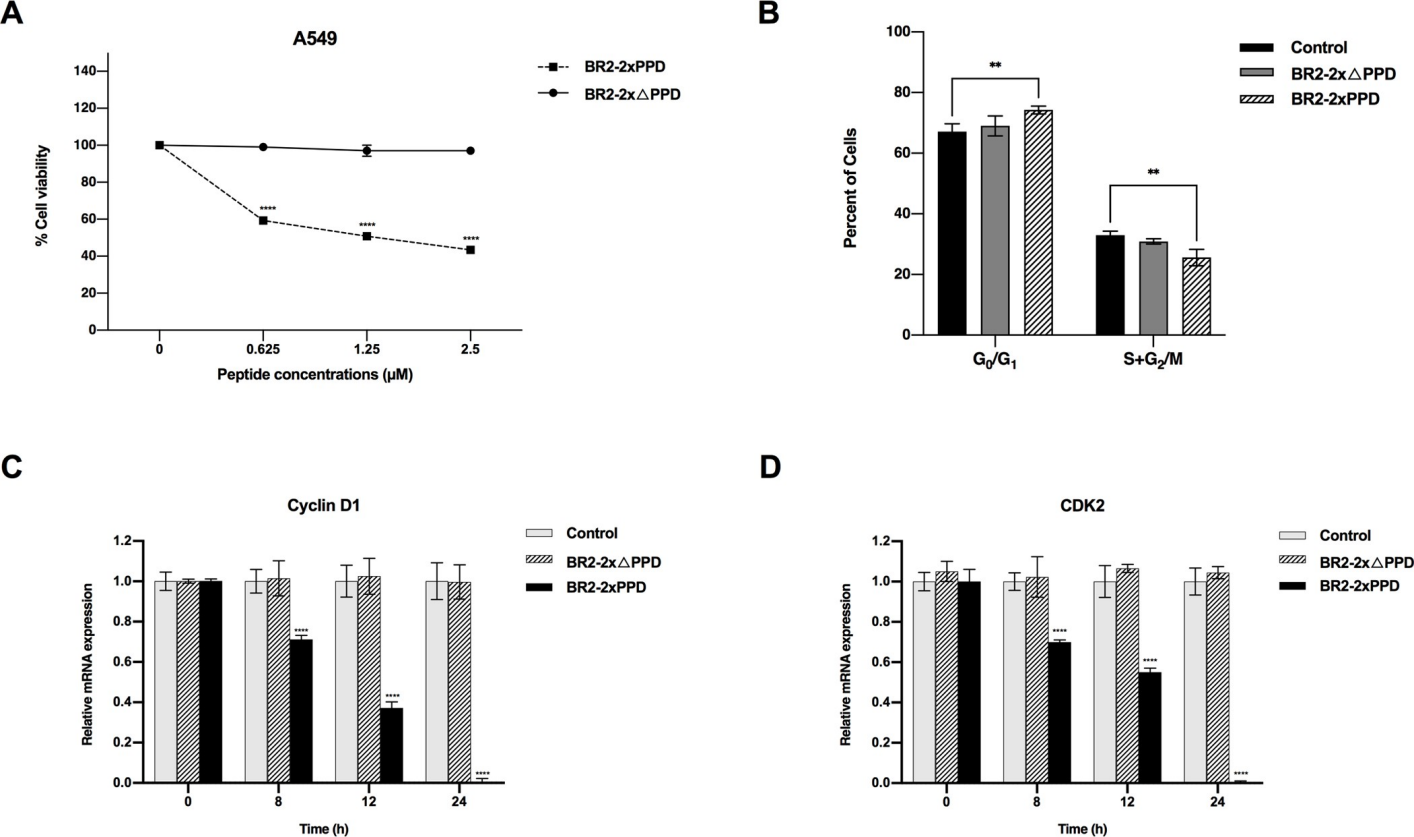
BR2-2xPPD peptide inhibited EGFR wild-type NSCLC growth. (A) Effect of BR2-2xPPD and BR2-2xΔPPD peptide on EGFR wild-type A549 cell proliferation. A549 cells were cultured in 10%FBS-RPMI plus 1% PenStrep at 3,000 cells/well. After 24 h, cells were treated with increasing dose of BR2-2xPPD or BR2-2xΔPPD (0, 0.625, 1.25 and 2.5 μM) for 24 h. Cell viability was performed by MTT assays. Value are represented as means ± SEM (n = 3). Percent cell viability of peptide treatments were normalized with DMSO (****p ≤ 0.0001). (B) Cell cycle distribution assessed by flow cytometry. A549 cells were treated with BR2-2xPPD or BR2-2xΔPPD peptide at concentration 2.5 μM in 10%FBS-RPMI for 24 h. Cells were collected and analyzed by flow cytometry as described in Materials and Methods. Bar graphs represent changes in the percentage of cells in G_0_/G_1_ and S+G_2_/M phase as compared with untreated cell (control). Results are shown as means ± SEM (n = 3) (**p ≤ 0.01). (C) Relative Cyclin D1 and (D) CDK2 mRNA expression. A549 were cultured with BR2-2xPPD peptides at a concentration of 2.5 μM in 10%FBS-RPMI for indicated time points shown in the x-axis. Total RNA was extracted and amplified by qRT-PCR. Values represent relative gene expression normalized with GAPDH. All data are reported as means ± SEM (n = 3) compared with control untreated cells (***p ≤ 0.001) (****p ≤ 0.0001).

Several drugs and chemotherapies are currently available for the treatment of NSCLC, including tyrosine kinase inhibitors (TKIs). However, the first generation of TKIs such as Gefitinib and Erlotinib are quite effective in inhibiting cells expressing mutated EGFR, but much less effective in the cells expressing wild-type EGFR [40]. Therefore, a novel alternative approach is needed for patients with NSCLC expressing wild-type EGFR. Our results demonstrated that the BR2-2xPPD peptide treatment of NSCLC inhibited EGF-mediated cell proliferation. We next tested the effect of the BR2-2xPPD peptide on NSCLC cell proliferation in medium supplemented with fetal bovine serum (FBS) without additional EGF. NSCLC expressing wild-type EGF, A549, were cultured with the presence of the BR2-2xPPD or BR2-2xΔPPD peptides at concentrations ranging from 0 to 2.5 µM for 24 h. Cell viability was assessed by MTT assay. The BR2-2xPPD peptide dose-dependently inhibited A549 cell proliferation with a maximum inhibition at 62% (±5%) at the highest dose while the presence of the BR2-2xΔPPD peptide had little to no effect on A549 cell proliferation (Fig 3A). We next determine whether the presence of the BR2-2xPPD affected cell cycle distribution of NSCLC. To assess the distribution of cells in different phases of the cell cycle, A549 were treated with the BR2-2xPPD or BR2-2xΔPPD for 24 h, and cell cycle were analyzed by flow cytometry. As shown in Fig 3B, the percentage of the G_0_/G_1_ phase was significantly increased while S+G_2_/M populations was significantly decreased in A549 treated with BR2-2xPPD compared to control, suggesting that the BR2-2xPPD peptide inhibited NSCLC growth in G_0_/G_1_ phase arrest, leading to a decrease in the percentage of proliferative cells in G2/M and S phases.

During cell cycle progression, cyclin D1 and CDK2 are critical cell cycle regulators involved in the G_0_/G_1_ to S phase transition and serve as theraputic targets in several types of cancers, including NSCLC [41, 42]. Our results suggested that BR2-2xPPD altered cell cycle progression. We next examined how BR2-2xPPD peptide treatment affected cyclin D1 and CDK2 expression. A549 were treated with BR2-2xPPD 2.5 µM at various time points. Cyclin D1 and CDK2 mRNA levels were quantitated by real-time PCR, using GAPDH as an internal control. Cells treated with BR2-2xPPD exhibited a significant decrease in cyclin D1 and CDK2 gene expression as compared to those of control untreated cells at all time points tested (Fig 3C and 3D). These data demonstrated that the BR2-2xPPD mediated cell cycle arrest and displayed antiproliferative activity resulting in a significant (57±1%) inhibition of proliferation of NSCLC expressing wild-type EGFR, suggesting that the BR2-2xPPD peptide could be further developed as an alternative new treatment for NSCLC expressing wild-type EGFR in the future.

### Treatment of the BR2-2xPPD peptide could enhanced growth inhibitory effects of Tyrosine Kinase Inhibitors (TKIs) in NSCLC harboring EGFR mutation

**Fig 4:**
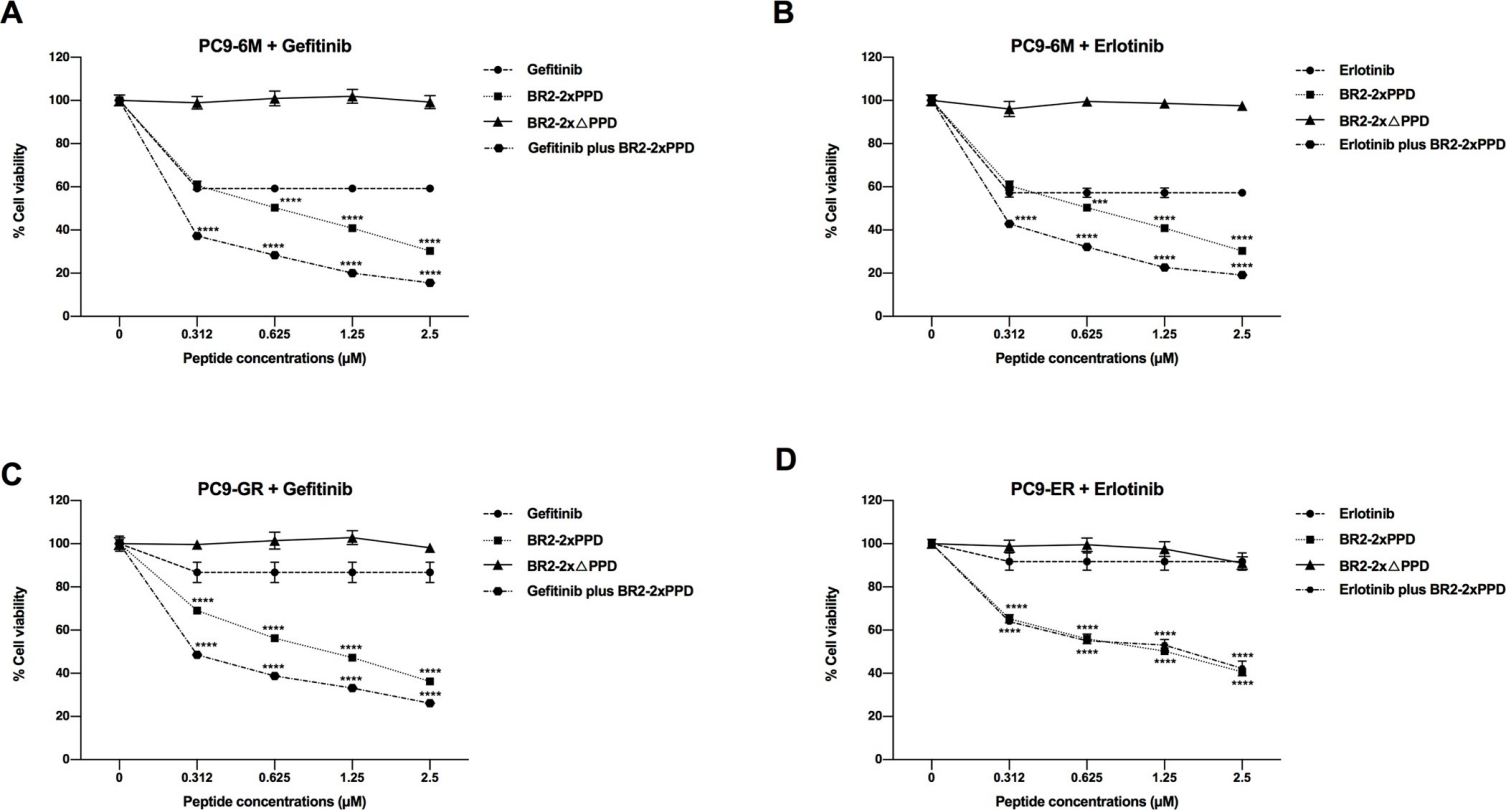
Effects of EGFR-TKIs and BR2-2xPPD peptide combination treatment in NSCLC harboring EGFR mutation. **4A & 4B** The parental PC9-6M cells expressing EGFR with exon 19 deletion, were treated with 0.1 µM Gefitinib (A) or Erlotinib (B) alone or combined with an increasing dose of BR2-2xPPD or BR2-2xΔPPD peptide (0–2.5 μM) for 72 h. **4C & 4D** Gefitinib or Erlotinib-resistant PC9, PC9-GR (C) or PC9-ER (D), were treated with 0.1 µM Gefitinib (C) or Erlotinib (D) alone or combined with an increasing dose of BR2-2xPPD or BR2-2xΔPPD-peptide (0–2.5 μM) for 72 h. Cells were analyzed for cell viability by MTT assays. Graphs represent percent cell viability of PC9-6M, PC9-GR or PC9-ER cells after indicated treatments normalized to cells treated with DMSO (control). Values are shown as means ± SEM in triplicate experiments (n = 3) and **** denotes p ≤ 0.0001.

The activating mutation of EGFR is frequently found in 5–20% of NSCLC patients. NSCLC tumors of patients who respond to the first-generation EGFR-TKIs often express mutated EGFR [43, 44]. However, most patients (50–60%) eventually develop acquired resistance to EGFR-TKIs after 12 months of initiating TKI treatment. Various approaches, such as new compounds or targeted therapies, have been used to prolong patients’ survival. To determine if the PR-PPD inhibits the growth of TKI-resistant lung cancer cells, we tested whether the combination treatment of the BR2 containing PR-PPD peptide (BR2-2xPPD) and TKIs could be more effective than TKIs alone in inhibiting lung cancer cell proliferation. EGFR-TKI-sensitive (PC9-6M) lung adenocarcinoma cells were treated with 0.1 µM concentration of Gefitinib or Erlotinib alone compared or in combination with TKIs- with increasing concentration of BR2-2xPPD peptides (0–2.5 µM) for 72 h. In TKI-sensitive PC9-6M cells, Gefitinib and Erlotinib reduced cancer cell proliferation by 41% (±2%) and 43% (±2%) as compared to control (no treatment). The combination of the BR2-2xPPD and TKIs, Gefitinib or Erlotibnib, further reduced cancer cell proliferation by 85% (±1%) and 81% (±1%), respectively (Fig 4A and 4B), while in the presence of the mutant PPD peptides, BR2-2xΔPPD, had little to no effect on PC9-6M cell growth.

To test whether the BR2-2xPPD peptide could enhance the growth-inhibitory effect of TKIs, we used EGFR-TKI-resistant variant (PC9-GR and PC9-ER) cell lines. PC9 were cultured with increasing concentrations of Gefitinib or Erlotinib; 10 nM (2 months), 1 µM (2 months) and 5 µM (2 months) to generate the Gefitinib-and Erlotinib-resistant cell lines (PC9-GR and PC9-ER, respectively) [33, 45]. PC9-GR and PC9-ER were treated with 0.1 µM concentration of Gefitinib or Erlotinib alone, or in combination with TKIs, compared with increasing concentration of the BR2-2xPPD peptides (0–2.5 µM) for 72 h. As shown in Fig 4C and 4D, Gefitinib or Erlotinib alone weakly inhibited PC9-GR and PC9-ER cell proliferation and inhibited only 13% (±5%) and 8% (±4%) as compared to control untreated cells, respectively, and these results were similar to previous reports [33]. The treatment of the BR2-2xPPD peptides in Gefitinib-resistant PC9-GR cells dose-dependently inhibited cell proliferation with a maximum inhibition at 64% (±1%) of control untreated cells. Interestingly, the treatment of BR2-2xPPD peptides in combination with Gefitinib enhanced the Gefitinib growth inhibitory effects and showed a maximum inhibition at 74% (±1%) as compared to control untreated cells. For Erlotinib-resistant PC9-ER cells, the BR2-2xPPD peptide dose-dependently suppressed cell proliferation with a maximum inhibition at 59% (±1%) of control untreated cells (Fig 4D). The combination treatment of the BR2-2xPPD with Erlotinib showed the maximum inhibition at 58% (±3%) compared to control cells which did not show significant differences to BR2-2xPPD treatment alone. These data suggest that the BR2 peptide containing PR-PPD can enhance the growth inhibitor effect of Gefitinib, suggesting that combination treatment of BR2-2xPR-PPD peptides with Gefitinib may serve as a new perspective in NSCLC treatment.

## Discussion

Cancer constitutes a major cause of mortality in both men and women worldwide. Current treatments are available for cancers such as surgery, radiotherapy and chemotherapy. For NSCLC, the predominant type of lung cancer, surgical resection is the most effective treatment in the early stages of the disease; however, the recurrence rates after the surgical resection remain high [46]. Chemotherapy and radiotherapy are commonly used to help improve survival outcomes in recurrent patients with advanced and metastatic NSCLC [47]. Despite a reduction in the mortality rate for patients with advanced metastatic NSCLC with the current therapeutic regimens most patients frequently suffer from adverse side effects due to non-specific targeting of chemotherapy drugs. Current chemotherapeutic agents not only affect the rapidly dividing cancer cells but also the fast-growing normal healthy cells. Therefore, new therapeutics options focusing on novel strategies to selectively target cancer cells with minimal to no effect on normal cells are urgently needed.

It is well established that PR signaling plays essential roles in endocrine-related cancers such as breast, endometrium and ovary [48]. PR is expressed as two isoforms from a single gene, PR-A and PR-B [49]. Proteomic profiling supports that the differences in the proteome expression of individual PR-A and PR-B isoforms contributes to the differences in the biological actions of each isoform in breast cancer cells [50]. In addition to progestin-dependent transcriptional effects, liganded-PR can rapidly activate other signaling molecules through SH3-polyproline domain (PR-PPD) interactions [19]. Although both PR isoforms share the identical PPD sequence, only PR-B mediates c-Src/Ras/MAPK signaling in the cytoplasm [21]. Increasing evidence suggests that PR also has a potential role in non-endocrine tumors, including NSCLC [51, 52]. Clinical data shows that high expression levels of PR is correlated with better clinical outcome in NSCLC patients. Treatment with progesterone promotes the inhibition of NSCLC growth *in vitro* and *in vivo*, and PR could potentially be used as a prognostic marker in NSCLC patients [23, 53–55].

We previously showed that PR-B expression in A549 NSCLC significantly blunted EGF-induced-NSCLC cell growth [24]. In this study, we extended our investigation by expressing the PR-PPD peptides fused with a cell-penetrating peptide domain, BR2 (Fig 1A). Consistent with our previous study, both BR2-PPD and BR2-2xPPD peptides significantly inhibited EGF-induced A549 NSCLC cell proliferation, while the mutant PR-PPD peptide failed to affect A549 cell proliferation (Fig 2A and 2B). These data implicated a crucial role of a short PR-PPD peptide suppressing NSCLC cell growth [24], suggesting that PR expression can directly affect cell proliferation.

Importantly, our results suggest a crosstalk between PR extranuclear signaling and growth factor receptors, independent of progesterone, can play a role in NSCLC progression. A549 cells pre-treated with BR2-2xPPD before treating with EGF showed significant attenuation of MAPK activation (Fig 2C). Little to no inhibition of MAPK activation was observed in cells pre-treated with the mutant peptide, BR2-2xΔPPD (Fig 2D). Together, these data suggest that BR2-PRPPD peptides can inhibit EGF-mediated MAPK activation, leading to a decrease in A549 cell proliferation.

Upon growth factor receptor activation, Grb2 and Sos interaction is required to transduce a variety of downstream signaling molecules, including MAPK. Sos-derived peptides were previously designed to disrupt Grb2-Sos interaction [56, 57]. The peptidimer peptide containing two-repeats of the proline-rich sequence has been demonstrated to be more effective in inhibiting Grb2-Sos interaction and more potent in reducing MAP kinase activation than the monomer peptide with one copy of the proline-rich sequence [56]. Furthermore, treatment with a peptidimer was shown to serve as a Grb2-SH3 ligand to reduce the growth of HER2-positive breast cancer cells [58]. These results are consistent with our results, in which the BR2-2xPPD peptide, with two repeats of the PPD, was more effective in reducing A549 cell proliferation than the BR2-PPD peptide, with a single PPD. These results suggest that the PPD in these peptides share a similar polyproline type II (PPII) helices structure [35] and can efficiently block Grb2-Sos complexes through dual interactions of PR-PPD with both SH3 domains of Grb2.

The PR-PPD mediates rapid progestin-mediated c-Src kinase activation through directly binding to the c-Src-SH3 domain [19]. This PPD is a unique sequence present in PR and absent in all other nuclear hormone receptors, including the estrogen receptor (ER), androgen receptor (AR), glucocorticoid receptor (GR) and thyroid hormone receptor (TR) [19]. These proline-rich peptides are particularly useful in obstructing the PPD of Grb2 from binding to the SH3 domains. However, the inability of these protein sequences to enter cells and bind to cytoplasmic signaling molecules has limited the use of these peptides. In this study, we added a cell-penetrating peptide domain to the PR-PPD to promote peptide internalization, allowing the PR-PPD to enter cells and interfere with PPD-SH3 mediated signal transductions.

In recent years, a new class of drug delivery molecules such as cell-penetrating peptides or CPPs has been developed. The truncated HIV-1 Tat peptide has been described as the first CPP to mediate the cellular uptake of target molecules [59, 60]. However, several CPPs exhibit non-specific penetration or cytotoxic effects at high concentrations. More recently the tumor-specific, less toxic, cell-penetrating BR2 peptide was successfully developed and used as a drug transporter and tested in colorectal carcinoma and hepatocellular cancer *in vivo* models [36, 61]. In this study, we designed and expressed the anticancer BR2 containing the PR-PPD peptide from *P*. *pastoris* to target growth factor signaling pathways that requires PPD-SH3 interactions in NSCLC. *P*. *pastoris* is an attractive expression host which can grow and produce high level of peptides on simple media [62]. Compared with bacteria, *P*. *pastoris* has eukaryotic post-translational modifications and secretory expression which can be directly purified [31]. Our results demonstrated the ability of BR2 peptides to be internalized into NSCLC cancer cells, with little to no effect on normal cells (Fig 1E). Because it is a highly positively charged peptide, BR2 preferentially binds to negatively charged ganglioside on the cancer cell membrane and consequently internalizes into the cytoplasm by micropinocytosis [36]. In contrast, the cell membrane of most normal cells is neutral, resulting in low electrostatic interaction and inefficient internalization of BR2 through the cell membrane [36, 63]. Thus, the PR-PPD present in the BR2-2xPPD, once internalized, can directly compete with the PPD in SOS to bind to the Grb2-SH3 domains, leading to a suppression of Grb2-SOS signal transduction, a significant reduction in Erk1/2 activation and a decrease in NSCLC cell proliferation.

Tyrosine Kinase Inhibitors (TKIs), such as Gefitinib and Erlotinib, are used as targeted therapy for patients with NSCLC whose tumors overexpress EGFR. TKIs compete with ATP to bind to the ATP binding pocket of tyrosine kinases, resulting in a reduction in tyrosine kinase phosphorylation and a decrease in cell proliferation [64]. Meta-analysis studies suggest that EGFR-TKIs improves progression-free survival (PFS) in patients with advanced NSCLC expressing mutant EGFR whilst having no significant impact in NSCLC patients with NSCLC expressing wild-type EGFR [65–69]. The mechanism mediating resistance to EGFR-TKIs in wild-type EGFR is well characterized. A previous study suggests that the lower TKI binding affinity of the wild-type EGFR attributes to its TKIs insensitivity [70]. Therefore, there is a need to identify novel compounds that more effectively block or suppress wild-type EGFR signaling. In this study, we demonstrate that the addition of the BR2-2xPPD peptides to the currently available EGFR-TKIs significantly improves TKI-mediated inhibition of NSCLC cell proliferation. Furthermore, our results show that a decrease in NSCLC by BR2-2xPPD is mediated, in part, through cell cycle G_0_/G_1_ arrest and a reduction in cyclin D1 and CDK2 expression in A549 NSCLC expressing wild-type EGFR (Fig 3). Cyclin D1 is a critical regulator in cell cycle, required for G_1_ to S phase progression while CDK2 helps promote DNA replication prior to cell cycle progression into the G2/S phase [21, 22, 71]. Cyclin D1 has previously been described as a target gene in progestin-mediated Src kinase signaling pathways. The BR2-2xPPD peptide treatment of NSCLC with wild-type EGFR effectively induced cell cycle arrest and decreased numbers of proliferative cells, while EGFR-TKIs, including Gefitinib and Erlotinib, failed to inhibit cell proliferation of NSCLC with wild-type EGFR (Fig 3). Together, these results suggest that the BR2-2xPPD peptide is an attractive molecule that can potentially be developed as a targeted therapeutic agent to help treat NSCLC harboring wild-type EGFR.

Constitutive hyperactivation of the MAPK cascade is frequently found in cells expressing activated EGFR mutants where the kinase domain is constitutively activated independent of EGF binding. Exon 19 deletion and the L858R point mutation are the most common activating mutations and are often associated with sensitivity to TKIs, including Gefitinib and Erlotinib [45, 72]. Although NSCLC patients whose tumors bear the EGFR mutation or deletion often show better clinical outcomes to EGFR-TKIs treatments, a large proportions of these patients (50–60%) eventually develop acquired resistance to TKIs caused by a secondary mutation, such as the T790M mutation [73–75]. The EGFR-T790M gatekeeper mutation is a substitution mutation inside the ATP binding cleft leading to the low-affinity binding of TKIs to EGFR [76]. Administration of TKIs at high concentrations can cause adverse side effects in NSCLC patients, such as skin rash and diarrhea. Therefore, ability to increase tumor sensitivity to TKI could be an attractive alternative therapeutic option benefiting NSCLC patients.

In this study, we demonstrated that BR2-2xPPD peptide treatment increases sensitivity to TKI in both TKI-sensitive PC9-6M cells and Gefitinib-resistant PC9-GR cells (Fig 4A–4C). Both PC9-6M and PC9-GR cells express a typical EGFR mutation in exon 19 (Fig 4A–4C). Here we show that the BR2-2xPPD peptide significantly suppresses Erlotinib-resistant PC9-ER cell proliferation (Fig 4D). However, the percentage inhibition of PC9-ER cell proliferation in cells treated with the BR2-2xPPD peptide alone was not significantly difference from those treated with the BR2-2xPPD peptide in combination with Erlotinib. Previous study suggested that the EFGR harboring exon 19 deletion, L858R mutation can acquire a secondary T790M mutation, making it insensitive to TKI. Thus, it is possible that differences in EGFR mutation may contribute to PC9-ER TKI insensitivity [33]. In addition, in TKIs-sensitive cells, TKI can effectively reduce Erk1/2 and Akt phosphorylation, leading to NSCLC growth inhibition [77], while, in TKI-resistant cells, growth factor receptors or other growth promoting genes are often activated and serve as alternative pathways stimulating cancer cell survival [78]. Thus, it is also possible that PC9-ER cells bear an activated EGFR mutant along with activation of other signaling pathways that are dependent on PPD-SH3 interactions for signal transduction, making PC9-ER insensitive to Erlotinib treatment but sensitive to the BR2-2xPPD peptide. Several studies have found that tumors with acquired resistance to Gefitinib and Erlotinib with or without the T790M mutation exhibit amplification of mesenchymal-epithelial transition factor (MET) [79–82]. MET is a heterodimer tyrosine kinase receptor which requires PPD-SH3 interaction for downstream signaling activation and its dysregulation could induce EGFR-TKIs resistance by activating ERBB3 signaling [83, 84]. Therefore, the combination of EGFR-TKIs and drugs targeting others signaling pathways such as the BR2-2xPPD peptide could be an effective strategy to overcome TKI resistance [85, 86].

Our results demonstrate the potential role of BR2, a small cell penetrating peptide specifically target cancer cells without damaging normal cells. Intracellular delivery of the PR-PPD by BR2 suppressed cell proliferation of NSCLC bearing either the wild-type or mutant EGFR. Treatment with the BR2-2xPPD peptide enhanced cytotoxic effects in acquired EGFR-TKI resistant lung cancer cells. Further studies will be needed to investigate the antitumor activity of the BR2-2xPPD peptide *in vivo*.

Altogether, our data establishes a proof of concept that a cancer cell-specific CPP in combination with the PR-PPD could serve as a novel therapeutic agent to suppress NSCLC growth. The BR2-2xPPD peptide may represent a new class of drugs for the treatment of NSCLCs by interfering with intracellular SH3-mediated signaling transduction.

## Supporting information

S1 File (DOCX)

S1 Raw images (PDF)
