# Supplementary material for: Cell-penetrating peptides containing the progesterone receptor polyproline domain inhibits EGF signaling and cell proliferation in lung cancer cells
Source: PLoS One. 2022 Mar 2;17(3):e0264717. doi: 10.1371/journal.pone.0264717 (PMC8890653; doi:10.1371/journal.pone.0264717)
Supplement: S1 File — (DOCX) [file pone.0264717.s001.docx]

Methods

Peptide Production from *Pichia pastoris*

Yeast transformation and DNA extraction

Preparation of competent yeast

Fresh *Pichia pastoris* strain KM71H single colony was inoculated in 10 ml YPD broth and cultured with shaking at 30°C, 250 rpm overnight. After 24 h, pipetted 4 ml from yeast cultured to 200 ml YPD (OD_600_ ~ 0.1) and growth with shaking for 3 h until reach an OD_600_ to ~1. Yeast cells were transferred to 50 ml conical tubes and harvested by centrifugation at 2000xg for 5 mins at 4°C. Resuspended yeast pellet in 10 ml YPD broth and 2 ml 1M HEPES (Bio-Basic, Canada) buffer pH.8. Gently mixed the cells with 250 µl fresh 1M DTT (Sigma Aldrich, USA) and incubated at 30°C for 15 mins and then placed the cells in ice-cold water for 5 mins. Harvested cells by centrifugation at 2000xg for 5 mins at 4°C, washed 2 times with 25 ml cold sterile water and centrifuged. Washed cells in 10 ml of cold 1M D-sorbitol (Bio-Basic, Canada) 2 times and resuspended yeast pellet in 50 µl of cold 1M D-sorbitol (total volume 100 µl). Cells were used immediately or stored at -80°C.

**Yeast transformation**

Total plasmid DNA 15 µg were digested with Sac *I* (New England Biolab, USA) and a complete linearization was analyzed by gel electrophoresis compared to undigested plasmid. The digested DNA was purified using QIAquick^®^ Gel/PCR extraction kit (Qiagen, Germany) and eluted in 15 µl DEPC. Total DNA was measured by Nanodrop. Purified 5-10 µg plasmid DNA in 10 µl was mixed to 40 µl of competent yeast and transferred into prechilled 2-mm gap cuvette on ice. Pulsed cells with high voltage (2.8 kV) and immediately added 500 µl of cold 1M D-sorbitol. The solution was transferred to a microcentrifuge tube and incubated at 30°C for 1 h. Added 500 µl YPD broth and incubated with shaking at 30°C, 200 rpm for 1 h. and then spread 300 µl on YPD plate containing 100 mg/ml zeocin and incubated at 30°C at least 2 days.

**Yeast Genomic extraction and PCR analysis**

To determine the recombinant gene has integrated into *P. pastoris* KM71H, genomic DNA was isolated from *Pichia* clone using YeaStar Genomic DNA kit (Zymo Research, USA). After colonies formed on YPD plate, 3-5 colonies were streaked for single colony at 30°C for 3 days. Cells were growth in YPD medium with shaking at 30°C, 250 rpm overnight. Yeast were harvested by centrifugation at 500xg for 2 min and pellet were resuspended in 120 µl of YD digestion buffer with 5 µl of R-Zymolase (Rnase A+Zymolase) by vortexing and incubated at 37°C for 1 h. Added 120 µl of YD lysis buffer and mixed by vortexing for 10 sec. Then, chloroform 250 µl was added and mixed for 1 min. The supernatant was separated by centrifugation at 13,000 rpm for 2 mins, then transferred to Zymo-spin III column and centrifuged for 1 min. Added 300 µl of DNA wash buffer and centrifuged for 1 min 2 times. Placed Zymo-spin III column to a new microcentrifuge tube and eluted DNA in 30 µl of DEPC. Total DNA was measured by Nanodrop. Yeast genomic DNA were extracted (Zymo research, USA) and further identified by PCR using 5’AOX (GACTGGTTCCAATTGACAAGC) and 3’AOX (GCAAATGGCATTCTGACATCC) primers. PCR products were analyzed by electrophoresis compared with P. pastoris containing empty pPICZαA.

Peptide expression and purification

**Peptide expression**

To prepare a starter culture, fresh single colonies of *P. pastoris* containing the CPP-peptide expression plasmid were inoculated into 10 ml of BMGY-buffered glycerol-complex medium and growth overnight at 30°C with shaking at 280 rpm until the OD_600_ reached 2-6. Then 10% of the starter culture was inoculated in 100 ml of BMGY medium in a 250 ml baffle flask for 24 h. Cells were harvested by centrifugation at 5,000xg for 5 min at room temperature. The supernatant was discarded. Yeast pellet was resuspended in 10 ml of Buffer Methanol-complex Medium (BMMY) containing 0.5% final concentration of methanol to induce protein expression. Cells were continuously incubated with shaking at 30°C, 280 rpm for 24 h. The cultured medium was collected by centrifugation at 5,000xg, 4°C for 20 mins and stored at -80°C.

**Peptide purification and dialysis**

The 6xHis-tag recombinant peptides were purified by nickel affinity chromatography (Histrap Fast Flow column, GE) using AKTA start. To prepare purification system, the HisTrap column was cleaned with sterile deionized water and equilibrated with binding buffer (20 mM Sodium Phosphate buffer pH 7.4, 0.5 M NaCl and 5 mM imidazole). Filtered supernatant was mixed with binding buffer in 1:2 ratio and loaded into column by pump injection. After sample loading, the HisTrap column was loaded with binding buffer for 5 column volumes to wash the unbound proteins. Histidine-tagged peptide was eluted by elution buffer (20 mM Sodium Phosphate buffer pH 7.4, 0.5 M NaCl and 500 mM imidazole) and collected by fraction collector. The expected fractions were pooled and then dialyzed to remove the excess salt. Peptide solution was transferred to 3.5 MWCO SnakeSkin Dialysis Tubing (Bio-Rad, Hercules, USA). Dialysis tubing was placed in 0.01M PBS pH 7.4 buffer with stirring on magnetic stirrer plate at 4°C for 3 h. Changed the dialysis buffer and dialyzed for twice more. Peptide concentrations were determined by Bradford assay (Bio-Rad, Hercules, USA).
