# Supplementary figures and images for: Cell-penetrating peptides containing the progesterone receptor polyproline domain inhibits EGF signaling and cell proliferation in lung cancer cells
Source: PLoS One. 2022 Mar 2;17(3):e0264717. doi: 10.1371/journal.pone.0264717 (PMC8890653; doi:10.1371/journal.pone.0264717)

Figure 1C

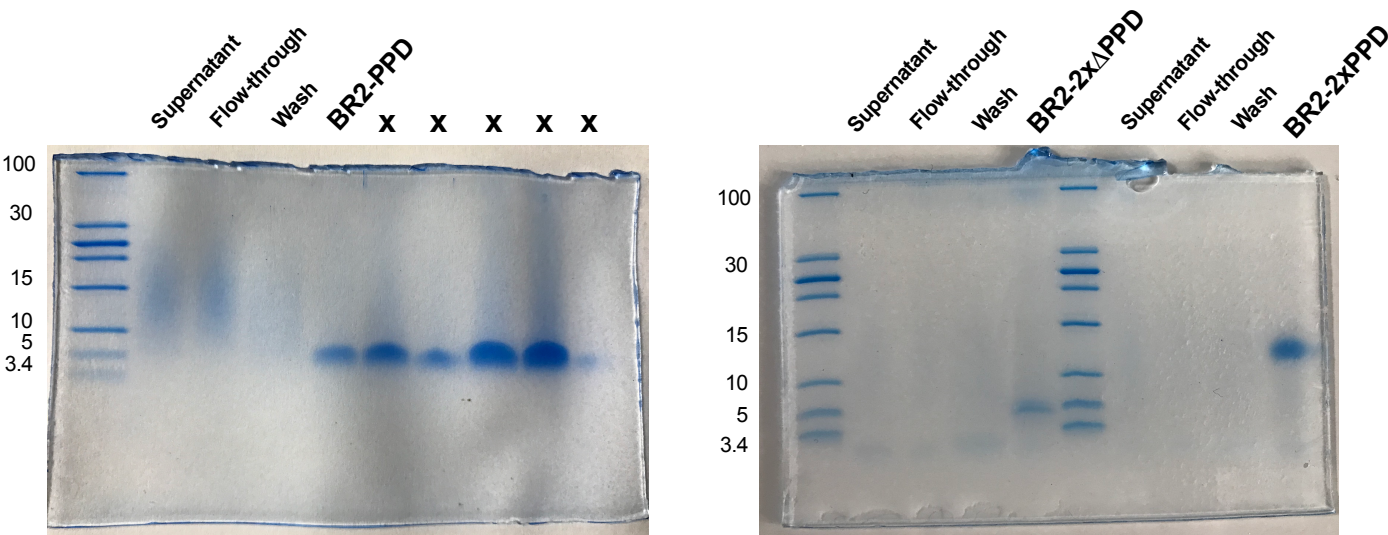

Figure 1D

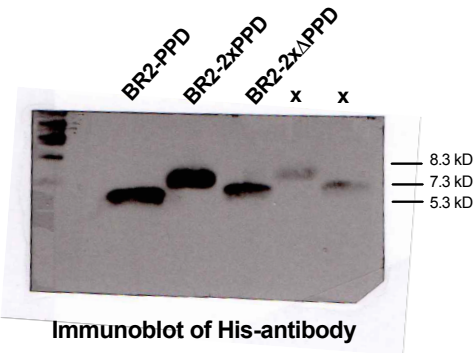

Figure 2C

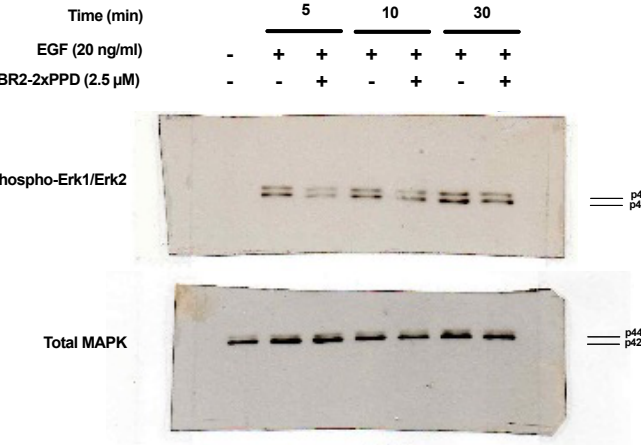

Figure 2D

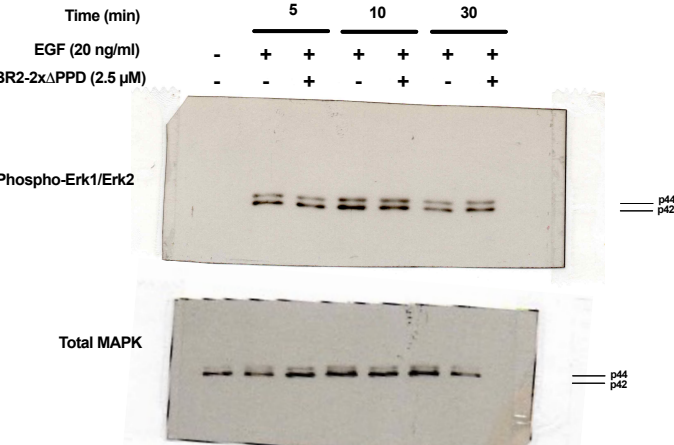

Supplement: S1 Raw images — (PDF) [file pone.0264717.s002.pdf]
